# Compound Fracture

**Published:** 1828-05

**Authors:** 


### COMPOUND FRACTURE.

### *Cases of Compound Fracture, treated at the* Royal Infirmary, Glasgow.

The melancholy terminations of those cases of compound fracture which the young practitioner has witnessed under the usual hospital treatment, naturally induce him to form the general resolution of amputating almost indiscriminately. He knows well that, whenever a case is presented to him, instant decision becomes necessary, that he has it not in his power to watch the first symptoms of approaching gangrene, and then to arrest its progress by the knife; but that, before replacing the torn integuments, or adjusting the shattered ends of the bone, he must fix, and irrevocably fix, amputate now or never. When, added to this, there crowd upon his mind the dreadful suppurations and mortified limbs of the hospital, can it be wondered at that the knife appears to him the safest line of conduct?

Leeches, cold applications, general depletion, and antiphlogistic regimen, large soft poultices and frequent dressings, all seem soothing and mild, and calculated to allay irritation: but do they prove so? Do not fulness of vessels and effusion supervene, tension of the fasciæ, erysipelatous integuments, irritative fever, deep-seated abscess, and gangrene?

What is compound fracture?—A broken bone, with a wound or tear of the soft parts. Then why do such frightful consequences seldom or never follow either of these two injuries, when occurring singly?—Because, in either of these two accidents, the limb is firmly bandaged, the weakened and torn vessels thereby supported, injection below the fascia prevented, and, above all, nature in her operations not too much interfered with.

Case I.—Angus Cameron, æt. fifty, an engineer, was admitted on the 7th July. Whilst removing some stones near a steam-engine, the wheel of the engine caught his right forearm, and, in endeavouring to save the trunk of his body, the left forearm became entangled also. The right radius was fractured one-third up, and on the back of the forearm there was a wound nearly four inches long, through which fractured portions of bones could be felt. Left radius was also fractured two inches above the carpal extremity, the superior portion of bone was much displaced, inwards and backwards; and on front of forearm, one-third up, there was a wound one inch and a half long, through which the fractured bone could be felt. On the anterior aspect of the superior third of the left forearm, there were two small wounds, and the probe passed freely and extensively under the surrounding integuments. The muscles of both forearms were severely lacerated, and there was much infiltration of blood, particularly in the left forearm, where the integuments felt very tense.

The wounds were dressed with oiled caddis, the many-tailed bandage applied, and cushions with wooden splints, after the manner for simple fracture.

On the 12th (five days after admission) it is reported: "Complained of no pain of either arm till last night, when the right began to be troublesome. On removing the dressings, there appears to be no swelling of any consequence, and the caddis is only moistened with healthy pus. Splints have not been removed from left arm. Slight delirium last night, but is costive, and speaks of pain about præcordia."

Two days afterwards the right arm was again bared, and about four ounces of thick purulent matter were pressed from an abscess in the immediate neighbourhood of the wound. From this time forward the right arm was dressed every day, and a small opening in the bandages left to give exit to the pus.

It appears unnecessary to lengthen out the report by recording trifling daily changes, either on the state of the right arm or of the general health; but the progress of the left arm deserves particular notice.

Although at first the left arm was looked upon as the more seriously injured of the two, yet he never complained of pain in it, nor were the splints or dressings removed till the 8th of August, being one month and one day after admission. The following is the report of that date: "Makes no complaint today, except of slight pain about left elbow-joint. Splints and dressings have been removed for the first time. Little or no pus, no swelling nor tension, one of the wounds all but closed, and only slight superficial ulceration from pressure at the elbow-joint."

Notwithstanding these favorable and encouraging circumstances, unpleasant symptoms had before this date manifested themselves, and his ultimate recovery was despaired of. Most extensive sloughing took place over the sacrum; and on the 16th of August he expired.

*Dissection.*—Fracture of the left radius had united, and the soft parts appeared firm and healthy in every part of the forearm. In the right forearm, the inferior portion of the fractured radius was split through its whole length. The superior and inferior portions of the bone at the seat of fracture were bare to the extent of two inches, and there was not the slightest appearance of an attempt at union.

Case II.—John Durrough, æt. thirty-two, a labourer, was admitted on the 16th July. A scaffold, two stories high, supporting a quantity of stones, on which he was standing, gave way. Some of the stones struck the lower jaw, the left ankle, and left hip, inflicting wounds on the two former. The lower jaw was fractured. The distal extremity of the left tibia was fractured transversely about an inch above its articulating surface. The fibula was fractured obliquely immediately above the malleolus; and the outside of the joint was laid open, anterior and posterior to the extensor tendons. On the outside of the ankle there was a wound of the integuments six inches long: it commenced at the extremity of the malleolus, and ran upwards in the course of the fibula, forming a flap, three inches broad from before backwards. On the inside of the ankle, there was another wound of the integuments: it commenced anterior to the inner malleolus, and ran transversely inwards and backwards around the ankle, communicating with the longitudinal wound on the outside of the ankle, both internal and external to the tendo Achillis. Along the outside of the joint there were many bony particles, which had been detached from the extremities of the tibia and fibula. The ankle-joint was but very slightly displaced. The large arteries, and all the tendons around the joint, had escaped, and were covered by the fascia; and the internal malleolus was not divided, nor were the ligaments injured on this side of the joint.

Pledgets of oiled caddis were placed over the wounds at the ankle, and fixed by adhesive plaster, and the limb put up after the manner for simple fracture. Neither splints nor dressings were removed till the 27th, being eleven days after admission. A little healthy pus was found on the wounds, which had begun to put on the appearance of healthy granulating sores. There was no redness, tension, or pain on pressure, at any part of limb, which was put up again in the same manner.

August 2d.—Splints and dressings have just been removed for the second time: wound has a granulating and healthy appearance. About half an ounce of pus, thick and healthy, and without fœtor, discharged. No redness nor tension. Limb can be freely moved about, for the application of bandages.

24th.—Two days ago had rigors, and an enlarged gland was observed in groin. Some erysipelatous redness on fore part of ankle appeared yesterday, and inflamed absorbents about knee-joint. Leeches were applied, and the pain and fever have diminished.

25th.—Redness increasing about knee-joint. Leeches were repeated this morning, with relief. Splints have still been kept on leg.

Sept. 1st.—Redness of thigh gone for some time, and feels no pain, except at outer part, and back of foot, where a small abscess has formed. A puncture has discharged half an ounce of pus.

10th.—Wound at inner ankle cicatrised; that at outer seems a healing sore; bones have reunited. Splints finally removed, being the fifth time they have been unbuckled since first application.

Straps and simple bandaging were used from this date, and on the 6th October he was dismissed cured.

Case III.—Thomas Poole, æt. eight, was admitted on the 17th of August. Whilst standing in the street, he was knocked down by the shaft of a cart, and one of the wheels went over his right leg and left foot. The toes of the left foot were fractured, and torn from their connexions to the metatarsus. The integuments of the right leg, from the superior third of the tibia, along the ridge of that bone to below the inner ankle, were detached from before backwards, and formed a thin flap above three inches broad. The fascia of the flexor muscles was destroyed, and the muscles separated from one another, but without loss of substance. The posterior tibial artery could not be felt. The tibia was fractured obliquely; the fracture communicated with the wound, and some small splinters were detached about four inches above the ankle. The foot felt cold.

All the five toes of the left foot were amputated, and a small portion of the distal end of the metatarsal bones clipped away. —The right leg was put up with straps, oiled caddis, bandage, and splints.

"27th (ten days after accident).—Bandages and dressings have just been removed for the first time from right leg. Little or no suppuration, no sloughing, and no tension of surrounding integuments. Surface of sore has a florid, granulating, and healthy appearance.

October 1st.—Fractured leg dressed again; pus healthy; granulations rather luxuriant and florid.

5th.—Third dressing of fractured leg: new skin beginning to shoot from every point. Bones united; granulations rather flabby, protracting the cure.

From this time forward it was treated as a common sore.*

---

### HERNIA.

* Dr. W. Young, in *Glasgow Med. Journal.*

